# Supplementary material for: Clinical utility and acceptability of a whole-hospital, pro-active electronic paediatric early warning system (the DETECT study): A prospective e-survey of parents and health professionals
Source: PLoS One. 2022 Sep 15;17(9):e0273666. doi: 10.1371/journal.pone.0273666 (PMC9477367; doi:10.1371/journal.pone.0273666)
Supplement: Attachment — Submitted filename: Rebuttal letter 13th June 2022.docx [file pone.0273666.s003.docx]

**Rebuttal to reviewer**

**Thank you for giving up your time to review our paper and for noting the one minor change we had missed in our previous revision.**

**The new text is presented in red.**

**Review Comments to the Author**

Please use the space provided to explain your answers to the questions above. You may also include additional comments for the author, including concerns about dual publication, research ethics, or publication ethics. (Please upload your review as an attachment if it exceeds 20,000 characters)

**Reviewer #1:**

**Minor edit required to conclusion as the children participants have been removed so need to be removed from conclusion:

"it is evident that the DETECT system has had success across three key groups of stakeholders: children, parents, and health professionals"**

Thank you for this comment, we have removed the reference to three stakeholder groups and the reference to children. The text now reads.

**(p25) …** **it is evident that the DETECT system has had success across two key groups of stakeholders: parents and health professionals.**
